# Supplementary material for: Microhabitat accessibility determines peptide substrate degradation by soil microbial community
Source: Microbiol Spectr. 2024 Dec 10;13(1):e01898-23. doi: 10.1128/spectrum.01898-23 (PMC11705811; doi:10.1128/spectrum.01898-23)
Supplement: Supplemental tables — Tables S1 to S4. [file spectrum.01898-23-s0001.docx]

### Supplementary material

|  | ***Model*** | ***df*** | ***AIC*** | ***BIC*** | ***Log Lik*** |  | ***Test*** | ***L. Ratio*** | ***p-value*** |
| --- | --- | --- | --- | --- | --- | --- | --- | --- | --- |
| *baseline* | 1 | 3 | 2217.928 | 2227.507 | -1105.964 |  |  |  |  |
| *Angles* | 2 | 5 | 2213.556 | 2229.520 | -1101.778 | 1 | vs 2 | 8.372250 | 0.0152 |
| *Angles + T. Order* | 3 | 6 | 2215.492 | 2234.650 | -1101.746 | 2 | vs 3 | 0.063176 | 0.8015 |
| *Interaction* | 4 | 8 | 2217.613 | 2243.157 | -1100.807 | 3 | vs 4 | 1.879087 | 0.3908 |

**Supplementary Table 1.** Output of the multi-level model fitting for the variable mean fluorescence intensity measured with MC fluorescence, with device as random effect and Angle and Turn order as fixed factors. Each step performs an ANOVA and compares the model with the previous model.

| ***contrast*** | ***estimate*** | ***SE*** | ***df*** | ***t.ratio*** | ***p.value*** |
| --- | --- | --- | --- | --- | --- |
| *45 - 90* | -53.41 | 19.9 | 175 | -2.689 | 0.0213 |
| *45 - 109* | -45.53 | 19.9 | 175 | -2.293 | 0.0595 |
| *90 - 109* | 7.88 | 19.9 | 175 | 0.397 | 0.9169 |

**Supplementary Table 2.** Contrasts of the multi-level model with the variable mean fluorescence intensity measured with MC fluorescence, with device as random effect and angle and turn order as fixed factors, using the Dunn’s method for adjusting confidence interval.

|  | ***Model*** | ***df*** | ***AIC*** | ***BIC*** | ***logLik*** | ***Test*** | ***L.Ratio*** | ***p-value*** |
| --- | --- | --- | --- | --- | --- | --- | --- | --- |
| *Baseline model* | 1 | 3 | 1242.339 | 1249.631 | -618.1695 |  |  |  |
| *Final Model* | 2 | 6 | 1215.864 | 1230.449 | -601.9322 | 1vs2 | 32.47469 | <.0001 |

**Supplementary Table 3.** Output of the multi-level model fitting for the variable mean fluorescence intensity measured with MC fluorescence, with device as random effect and maze type as fixed factor for the final model. Each step performs an ANOVA and compares the model with the previous model.

| ***contrast*** | ***estimate*** | ***SE*** | ***df*** | ***t.ratio*** | ***p.value*** |
| --- | --- | --- | --- | --- | --- |
| *F0 - F1* | -27.1 | 95.5 | 78 | -0.284 | 0.9920 |
| *F0 - F4* | -239.7 | 95.5 | 78 | -2.509 | 0.0663 |
| *F0 - F5* | -519.7 | 95.5 | 78 | -5.440 | <.0001 |
| *F1 - F4* | -212.6 | 95.5 | 78 | -2.225 | 0.1254 |
| *F1 - F5* | -492.6 | 95.5 | 78 | -5.156 | <.0001 |
| *F4 - F5* | -280.0 | 95.5 | 78 | -2.931 | 0.0225 |

**Supplementary Table 4.** Contrasts of the multi-level model with the variable mean fluorescence intensity measured with MC fluorescence, with device as random effect and maze type as fixed factor, using the Dunn’s method for adjusting confidence interval.
